# The review of innovative integration of *Kampo* medicine and Western medicine as personalized medicine at the first multidisciplinary pain center in Japan

**DOI:** 10.1186/1878-5085-5-10

**Published:** 2014-06-27

**Authors:** Young-Chang P Arai, Hiromichi Yasui, Hideya Isai, Takashi Kawai, Makoto Nishihara, Jun Sato, Tatsunori Ikemoto, Sinsuke Inoue, Takahiro Ushida

**Affiliations:** 1 Multidisciplinary Pain Center, Aichi Medical University, 21 Karimata, Nagakutecho, Aichigun, Aichi 480-1195, Japan

**Keywords:** *Kampo* medicine, Multidisciplinary pain center, Chronic and intractable pain

## Abstract

**Background:**

The Japanese medical system is unique because it is the only country in the world where Western medicine and traditional Japanese medicine including *Kampo* medicine, traditional Japanese herbal medicine, are used in our daily clinical practice. Pain is essentially an interactive psychophysiological behavior pattern. Thus, an interdisciplinary approach is often recommended in providing appropriate therapeutic care for the patients suffering from chronic and intractable pain. In addition, we have been prescribing Kampo medicines in combination with Western medicines as personalized medicine in order to treat patients with chronic pain at our pain center. The aim of our study was to conduct a survey on the current use and the effect of Kampo medicines in our multidisciplinary pain center.

**Methods:**

Retrospective analysis was performed on 221 out of 487 patients suffering from chronic pain.

**Results:**

The most frequent medical complaints for which Kampo medicines were prescribed were lower back/lower limb pain, neck/upper limb pain, various facial pains, headache/migraine, whiplash-associated disorder, and frozen shoulder. Kampo medicines were prescribed based on patient-centered Kampo diagnosis. Moreover, several Kampo medicines generally for the management of psychological symptoms were prescribed for about 70% of the patients. Pain improvement in the patients was categorized as follows: 26.3% with marked improvement, 12.7% with moderate improvement, 38.9% with some improvement, and 19.9% with no improvement.

**Conclusions:**

Two thirds of the chronic pain patients with the use of Kampo medicines combined with Western medicine experienced further pain improvements.

## Overview

Traditional, complementary, and alternative therapies are widely used and researched in the USA and Europe [1,2]. One of the underlying reasons for this is to reduce the high costs of health care. There is a national health insurance system which enables everyone in Japan to receive advanced health care at a low cost. Another characteristic of the health insurance system in Japan is that patients can access Western and *Kampo* medical care at the same time in the same medical institution [1,2]. Kampo, or traditional Japanese herbal medicine based on traditional Chinese herbal medicine, has been used for the treatment of not only acute but also chronic pain in Japan [3]. The Ministry of Health, Labor and Welfare of Japan, approves the use of 148 Kampo preparations (traditional herbal medicines) [4]. Currently, most Kampo preparations are prescribed as extract formulations of high quality. Moreover, physicians who have studied Western medicine and Kampo medicine practice Western and Kampo medical care in their daily clinical practice.

Pain is essentially an interactive psychophysiological behavior pattern, so an appreciation of the biopsychosocial model is essential for understanding and caring for patients with chronic pain. Thus, an interdisciplinary approach is often recommended and considered to be extremely relevant in providing appropriate therapeutic care for patients suffering from chronic and intractable pain [5]. Our center is the first multidisciplinary pain center established in Japan at July 2007. At our center, we have been prescribing Kampo extract formulations in combination with Western medicines in our daily clinical practice as an interdisciplinary approach in order to treat patients with chronic pain. However, to date, there have not been any reports on Kampo practice in multidisciplinary pain centers anywhere in the world because of the health insurance systems. We thus conducted a survey on the use of Kampo extract formulations and the effect of the formulations on patients with chronic pain in our multidisciplinary pain center.

## Methods

Retrospective analysis from August 2012 to July 2013 was performed on 487 patients suffering from chronic pain who visited the pain center of Aichi Medical University Hospital. All patients were referred from other hospitals to the pain center. Patients who were prescribed Kampo extract formulations were included.

After obtaining approval from the Ethics Committee of Aichi Medical University (a reference number, 13–097) and written informed consent, we routinely recorded demographics, symptoms, and course of pain in all patients. The intensity of pain was rated by the patients using a numerical rating scale (NRS) where 0 indicated no pain and 10 the greatest pain possible. All demographic and clinical data were extracted from medical records from August 2012 to March 2014 for the present study. In addition, patients were categorized as having marked improvement (≥60% improvement in NRS for pain compared to initial visit), moderate improvement (≥30% and <60% improvement in NRS compared to initial visit), some improvement (≥20% and <30% improvement in NRS compared to initial visit), and no improvement (<20% improvement in NRS compared to initial visit) depending on the state of pain improvement 6 months to 1 year after the initial visit. Moreover, patients who did not visit the hospital again even with an appointment for a follow-up visit and who visited the hospital for the purpose of receiving a second opinion were categorized separately.

## Results

We administered treatment after a medical conference attended by different types of professionals (anesthesiologists, orthopedists, psychiatrists, internists, dentists, nurses, physical therapists, and clinical psychotherapists). As required at the medical conference, we administered pharmacological (including Kampo medicine), physical, acupuncture, cognitive-behavioral, psychoanalytic, and psychological treatment. Kampo medicines were prescribed for 221 patients out of a total of 487 patients based on patient-centered Kampo diagnosis [3] while continuing Western medical care and the demographic characteristic data are presented in Table 1. Table 2 lists the most frequent medical complaints for which Kampo medicines were prescribed. Kampo medicines were prescribed to treat lower back/lower limb pain (36.6%, *n* = 81), neck/upper limb pain (13.1%, *n* = 29), various facial pains (13.1%, *n* = 29), headache/migraine (7.7%, *n* = 17), whiplash-associated disorder (5.9%, *n* = 13), and frozen shoulder (3.6%, *n* = 8). Pain improvement in all the patients who were prescribed Kampo medicines was categorized as follows: 26.3% with marked improvement, 12.7% with moderate improvement, 38.9% with some improvement, and 19.9% with no improvement. Table 3 shows Kampo therapy outcome of each complaint.

**Table 1:** Patient's characteristics

**Patient's characteristics**	**Values**
Age (years)	57 [13–89]
Sex (M/F)	79/142
Weight (kg)	56 [34–92]
Duration of pain (months)	58 [3–400]

Values are numbers or mean [range].

**Table 2:** Most frequent medical complaints for which Kampo medicines were prescribed

**Medical complaint**	**% (*n*)**
Lower back/lower limb pain	36.6 (81)
Neck/upper limb pain	13.1 (29)
Various facial pains	13.1 (29)
Headache/migraine	7.7 (17)
Whiplash*-*associated disorder	5.9 (13)
Frozen shoulder	3.6 (8)

**Table 3:** Duration of pain and Kampo therapy outcome

	**LB** ***/*** **LL pain**	**N** ***/*** **UL pain**	**Various facial pains**	**Headache** ***/*** **migraine**	**W** ***-*** **A disorder**	**Frozen shoulder**
Duration of pain (months)						
Median (range)	30 (3–360)	24 (3–360 )	24 (3–360)	36 (3–360)	12 (3–360)	18 (3–36)
Improvement	% (*n*)	% (*n*)	% (*n*)	% (*n*)	% (*n*)	% (*n*)
Marked	23.5 (19)	34.5 (10)	34.5 (10)	47.0 (8)	30.8 (4)	88.9 (7)
Moderate	17.3 (14)	20.6 (6)	17.2 (5)	0.0(0)	15.4 (2)	11.1 (1)
Some	23.5 (19)	13.7 (61)	10.3 (3)	12.8 (2)	7.9 (1)	0.0 (0)
No	28.4 (23)	17.2 (5)	30.8 (9)	17.6 (3)	30.8 (4)	0.0 (0)
Dropouts	4.8 (4)	10.3(3)	6.9 (2)	23.5 (4)	15.4 (2)	0.0 (0)

Of 221 new patients at Aichi Medical University Multidisciplinary Pain Center. LB/LL pain, lower back/lower limb pain; N/UL pain, neck/upper limb pain; W-A disorder, whiplash-associated disorder.

Table 4 lists the most frequently prescribed Kampo medicines for lower back/lower limb pain. We prescribed *Goshajinkigan* (22.2%, *n* = 18), *Shakuyakukanzoto* (17.3%, *n* = 14), *Yokukansan* (16.0%, *n* = 13), *Keishikajutsubuto* (14.8%, *n* = 12), *Hachimijiogan* (14.8%, *n* = 12), and *Juzentaihoto* (14.8%, *n* = 12) for the management of lower back/lower limb pain. Pain improvement in the patients who were prescribed Kampo medicines was categorized as follows (Table 3): 23.5% with marked improvement, 17.3% with moderate improvement, 23.5% with some improvement, and 28.4% with no improvement. In contrast, pain improvement in the patients who were not prescribed Kampo medicines (*n* = 136; median (range) of pain duration, 24 (3–320) months) was categorized as follows: 19.1% (*n* = 26) with marked improvement, 6.9% (*n* = 9) with moderate improvement, 12.5% (*n* = 17) with some improvement, and 25.0% (*n* = 34) with no improvement.

**Table 4:** Most frequently prescribed Kampo medicines

**Kampo medicine**	**% (*n*)**	**Ingredients (6, 8)**
Lower back/Lower limb pain		
Goshajinkigan	22.2 (18)	Rehmannia, Cornus, Dioscorea, Alisma, Hoelen, Moutan, Cinnamon, Aconite, Achyranthes, Plantago Jukujio
Shakuyakukanzoto	17*.*3 (14)	Glycyrrhizae radix, Peony, Liquorice
Yokukansan	16*.*0 (13)	Tang*-*kuei, Gambir, Cnidium, Atractylodes, Holen, Bupleurum, Liquorice
Keishikajutsubuto	14*.*8 (12)	Cinnamon, Hoelen, Moutan, Persica, Peony
Hachimijiogan	14*.*8 (12)	Rehmannia, Cornus, Dioscorea, Alisma, Hoelen, Moutan, Cinnamon, Aconite
Juzentaihoto	14*.*8 (12)	Ginseng*,* Astragalus, White atractylodes, Tang*-*kuei, Hoelen, Rehmannia, Cnidium, Peony, Cinnamon, Liquorice
Neck*/*Upper limb pain		
Keishibukuryogan	34*.*5 (10)	Cinnamon, Atractylodes, Aconite
Yokukansan	24*.*1 (7)	Tang*-*kuei, Gambir, Cnidium, Atractylodes, Holen, Bupleurum, Liquorice
Jidabokuippo	17*.*3 (5)	Cinnamon, Cnidium, Glycyrrhizae radix, Rhei rhizome, Quercus, Caryophylli, Nupharis rhizoma
Tokishakuyakusa	17*.*3 (5)	Tang*-*kuei, Cnidium, Peony, Hoelen, Atractylodes, Alisma
Kamishoyosan	13*.*8 (4)	Tang*-*kuei, Peony, Atractylodes, Hoelen, Bupleurum, Liquorice, Moutan, Gardenia, Ginger, Mentha
Kakkonto	13*.*8 (4)	Pueraria, Ma*-*huang, Ginger, Jujube, Cinnamon, Peony, Liquorice
Kososan	13*.*8 (4)	Cyperus, Perilla, Citrus, Ginger, Liquorice
Various facial pains		
Kamishoyosan	44*.*8 (13)	Tang ***-*** kuei, Peony, Atractylodes, Hoelen, Bupleurum, Liquorice, Moutan, Gardenia, Ginger, Mentha
Yokukansan	44.8 (13)	Tang-kuei, Gambir, Cnidium, Atractylodes, Holen, Bupleurum, Liquorice
Maobushisaishinto	20.7 (6)	Ma-huang, Asarum, Aconite
Keishibukuryogan	10.3 (3)	Cinnamon, Atractylodes, Aconite
Goreisan	10.3 (3)	Alisma, Polyporus, Hoelen, Atractylodes, Cinnamon
Headache/Migraine		
Keishibukuryogan	29.4 (5)	Cinnamon, Atractylodes, Aconite
Kamishoyosan	23.5 (4)	Tang-kuei, Peony, Atractylodes, Hoelen, Bupleurum, Liquorice, Moutan, Gardenia, Ginger, Mentha
Goshuyuto	23.5 (4)	Evodia, Ginseng, Ginger, Jujube
Jidabokuippo	23.5 (4)	Cinnamon, Cnidium, Glycyrrhizae radix, Rhei rhizome, Quercus cortex, Caryophylli, Nupharis Rhizoma
Whiplash-associated disorder		
Jidabokuippo	61.5 (8)	Cinnamon, Cnidium, Glycyrrhizae radix, Rhei rhizome, Quercus cortex, Caryophylli, Nupharis Rhizoma
Keishibukuryogan	38.5 (5)	Cinnamon, Atractylodes, Aconite
Tokishakuyakusan	23.1 (3)	Tang-kuei, Cnidium, Peony, Hoelen, Atractylodes, Alisma
Maobushisaishinto	15.4 (2)	Ma-huang, Asarum, Aconite
Yokukansan	15.4 (2)	Tang-kuei, Gambir, Cnidium, Atractylodes, Holen, Bupleurum, Liquorice
Frozen shoulder		
Nijutsuto	87.5 (7)	White and blue atractylodes, Hoelen, Citrus, Arisaema, Cyperus, Scute, Clematis, Chianghuo, Pinellia, Liquorice, Ginger
Keishikajutsubuto	37.5 (3)	Cinnamon, Hoelen, Moutan, Persica, Peony

For lower back/lower limb pain, neck/upper limb pain, various facial pains, headache/migraine, whiplash-associated disorder, and frozen shoulder.

Table 4 lists the most frequently prescribed Kampo medicines for neck/upper limb pain. We prescribed *Keishibukuryogan* (34.5%, *n* = 10)*,* Yokukansan (24.1%, *n* = 7), *Jidabokuippo* (17.3%, *n* = 5), *Tokishakuyakusa* (17.3%, *n* = 5), *Kamishoyosan* (13.8%, *n* = 4), *Kakkonto* (13.8%, *n* = 4), and *Kososan* (13.8%, *n* = 4) for the management of neck/upper limb pain. Pain improvement in the patients who were prescribed Kampo medicines was categorized as follows (Table 3): 34.5% with marked improvement, 20.6% with moderate improvement, 13.7% with some improvement, and 17.2% with no improvement. In contrast, pain improvement in the patients who were not prescribed Kampo medicines (*n* = 65; median (range) of pain duration, 24 (3–168) months) was categorized as follows (Table 3): 20.0% (*n* = 13) with marked improvement, 9.2% (*n* = 6) with moderate improvement, 13.8% (*n* = 9) with some improvement, and 26.2% (*n* = 17) with no improvement.

Table 4 lists the most frequently prescribed Kampo medicines for various facial pains. We prescribed Kamishoyosan (44.8%, *n* = 13)*,* Yokukansan (44.8%, *n* = 13), *Maobushisaishinto* (20.7%, *n* = 6), Keishibukuryogan (10.3%, *n* = 3), and *Goreisan* (10.3%, *n* = 3) for the management of various facial pains. Pain improvement in the patients was categorized as follows (Table 3): 34.5% with marked improvement, 17.2% with moderate improvement, 10.3% with some improvement, and 30.8% with no improvement.

Table 4 lists the most frequently prescribed Kampo medicines for headache/migraine. We prescribed Keishibukuryogan (29.4%, *n* = 5)*,* Kamishoyosan (23.5%, *n* = 4), *Goshuyuto* (23.5%, *n* = 4), and Jidabokuippo (23.5%, *n* = 4) for the management of headache/migraine. Pain improvement in the patients who were prescribed Kampo medicines was categorized as follows (Table 3): 47.0% with marked improvement, 12.8% with some improvement, and 17.6% with no improvement. In contrast, pain improvement in the patients who were not prescribed Kampo medicines (*n* = 20; median (range) of pain duration, 42 (3–240) months) was categorized as follows: 25.0% (*n* = 5) with marked improvement, 10.0% (*n* = 2) with moderate improvement, 10.0% (*n* = 2) with some improvement, and 15.0% (*n* = 3) with no improvement.

Table 4 lists the most frequently prescribed Kampo medicines for whiplash-associated disorder. We prescribed Jidabokuippo (61.5%, *n* = 8), Keishibukuryogan (38.5%, *n* = 5), Tokishakuyakusa (23.1%, *n* = 3), Maobushisaishinto (15.4%, *n* = 2), and Yokukansan (15.4%, *n* = 2) for the management of whiplash-associated disorder. Pain improvement in the patients who were prescribed Kampo medicines was categorized as follows (Table 3): 30.8% with marked improvement, 15.4% with moderate improvement, 7.9% with some improvement, and 30.8% with no improvement. In contrast, pain improvement in the patients who were not prescribed Kampo medicines (*n* = 12; median (range) of pain duration, 18 (3–144) months) was categorized as follows: 8.3% (*n* = 1) with marked improvement, 16.7% (*n* = 2) with some improvement, and 33.5% (*n* = 17) with no improvement.

Table 4 lists the most frequently prescribed Kampo medicines for frozen shoulder. We prescribed *Nijutsuto* (87.5%, *n* = 7) and Keishikajutsubuto (37.5%, *n* = 3) for the management of frozen shoulder. Pain improvement in the patients was categorized as follows (Table 3): 88.9% with marked improvement and 11.1% with moderate improvement.

## Expert recommendations

Since the 1970s, great attention has been given to traditional, complementary, and alternative therapies around the world [1,2]. One of the reasons for the attention is the limited effectiveness of biomedicine for the treatment of chronic diseases. Based on the Japanese health insurance system, we can use Western medicine and traditional Japanese medicine including Kampo medicine in our daily clinical practice at the same time in the same medical institution [1,2,4]. Furthermore, Kampo has been used for the treatment of chronic pain in Japan from ancient times to the present [3,6,7].

The results of the present survey showed that Kampo medicines were prescribed to treat lower back/lower limb pain (36.6%, *n* = 81), neck/upper limb pain (13.1%, *n* = 29), various facial pains (13.1%, *n* = 29), headache/migraine (7.7%, *n* = 17), whiplash-associated disorder (5.9%, *n* = 13), and frozen shoulder (3.6%, *n* = 8). In fact, we had these cases in the same order in our center and Kampo medicines were prescribed when treatment with Western medicine alone was insufficient. And this survey shows that the use of Kampo medicines combined with Western medicine as an interdisciplinary approach provided some improvements for two thirds of these patients refractory to Western medicine alone.

Goshajinkigan, Shakuyakukanzoto, Yokukansan, Keishikajutsubuto, Hachimijiogan, and Juzentaihoto were the most frequently prescribed Kampo medicines for lower back/lower limb pain. Goshajinkigan, Shakuyakukanzoto, Keishikajutsubuto, and Hachimijiogan have been used since ancient times to treat melosalgia, low back pain, and numbness [8-11]. Yokukansan has been used to treat excitability, depression, and excessive muscle tension [8,9,12].

Keishibukuryogan, Yokukansan, Jidabokuippo, Tokishakuyakusa, Kamishoyosan, Kakkonto, and Kososan were the most frequently prescribed Kampo medicines for neck/upper limb pain. Keishibukuryogan, Yokukansan, Tokishakuyakusa, Kamishoyosan, Kakkonto, and Kososan have been used for the management of headache and chronic neck pain [8,9,13,14].

Kamishoyosan, Yokukansan, Maobushisaishinto, Keishibukuryogan, and Goreisan were the most frequently prescribed Kampo medicines for various facial pains. Keishibukuryogan, Kamishoyosan and Yokukansan have been prescribed to manage emotional distress, headache, and clenching or grinding of the teeth [8,9,12-14].

Keishibukuryogan, Kamishoyosan, Goshuyuto, and Jidabokuippo were the most frequently prescribed Kampo medicines for headache/migraine. Goshuyuto has been used since ancient times to treat a very severe headache accompanied by vomiting [8,9,15]. Moreover, Keishibukuryogan and Kamishoyosan have been prescribed for the treatment of emotional distress, a heavy feeling in the head and headache [8,9,13,14].

Jidabokuippo, Keishibukuryogan, Tokishakuyakusa, Maobushisaishinto, and Yokukansan were the most frequently prescribed Kampo medicines for whiplash-associated disorder. Jidabokuippo and Keishibukuryogan have been prescribed for the management of sprains and trauma or bruises [6,8,9,14].

Nijutsuto and Keishikajutsubuto were the most frequently prescribed Kampo medicines for frozen shoulder. Nijutsuto has been the first-line Kampo medicine for the treatment of frozen shoulder [8,9]. Keishikajutsubuto has been used to treat arthritis [9].

The interesting thing about the present survey is that we usually prescribed Yokukansan, Kamishoyosan, and Kososan, which have generally been prescribed for the management of psychological symptoms [3,12,13,16,17]. Yokukansan and Kamishoyosan have anxiolytic effects [12,13,17] and especially Yokukansan which is known to exert these effects via serotonin receptors [12]. Pain is essentially an interactive psychophysiological behavioral pattern [5]. Our previous study also showed that about 70% of patients were moderate to high psychopathological patients [3]. We thus postulated that Yokukansan, Kamishoyosan, and Kososan were used for psychopathological patients suffering from chronic pain at our center.

Since pain is an interactive psychophysiological behavioral pattern, it is important for medical staffs to recognize the biopsychosocial model when understanding and caring for patients with chronic pain. Thus, an interdisciplinary approach is often recommended and considered to be extremely relevant in providing appropriate therapeutic care for patients with chronic and intractable pain [5]. That is, inter-, multidisciplinary integrated approach is needed for patients with chronic and intractable pain as personalized medicine. Kampo has been used for the treatment of chronic pain in Japan from ancient times to the present [3,6,7]. Also, Kampo medicines have been prescribed based on patient-centered Kampo diagnosis. Thus, we postulate that Kampo could be part of personalized medicine. Accordingly, we have been prescribing Kampo extract formulations in combination with Western medicines in our daily clinical practice as personalized medicine in order to treat patients with chronic pain at our center. And we expect that the number of physicians who use Kampo in this way will increase soon.

There are merits and demerits for the clinical application of Kampo medicine, when compared with Western medicine. A lot of patients value Kampo medicine as a holistic, body harmonizing treatment and as a stimulant for self-healing without severe side effects [1]. In contrast, some patients suffering from chronic pain are likely to be so dependent and tend to be reluctant to receive the treatments based on its holistic, self-healing philosophy.

There are several limitations of the study. The present report is a retrospective and nonrandomized control analysis of Kampo treatment and thus lacks the reproducibility. Since we have to clarify the specific kind of pain for personalized approach and detailed points for multidisciplinary integration, we need prospective and comparative study that might be more appropriate to support predictive, preventive, personalized value, and the reproducibility of Kampo treatment, thereby obtaining solid evidence of Kampo medicine in personalized pain management algorithm.

### Conclusion

The retrospective analysis on 221 out of 487 patients suffering from chronic pain who visited the pain center of Aichi Medical University Hospital showed that the most frequent medical complaints for which Kampo medicines were prescribed were lower back/lower limb pain, neck/upper limb pain, various facial pains, headache/migraine, whiplash-associated disorder, and frozen shoulder. Two thirds of the chronic pain patients with the use of Kampo medicines combined with Western medicine experienced further pain improvements. Moreover, we usually used several Kampo medicines generally prescribed for the management of psychological symptoms.

## Competing interests

The authors declare that they have no competing interests.

## Authors’ contributions

Y-CPA conceived of the study, participated in its study, and conducted all experiments. All authors conducted the acquisition of data. All authors helped to draft the manuscript. All authors read and approved the final manuscript.

## References

[B1] Hottenbacher L Weißhuhn TE Watanabe K Seki T Ostermann J Witt CM Opinions on Kampo and reasons for using it–results from a cross-sectional survey in three Japanese clinics BMC Complement Altern Med 2013 13 108 doi:10.1186/1472-6882-13-108 23680097 10.1186/1472-6882-13-108 PMC3669030

[B2] Moschik EC Mercado C Yoshino T Matsuura K Watanabe K Usage and attitudes of physicians in Japan concerning traditional Japanese medicine (kampo medicine): a descriptive evaluation of a representative questionnaire-based survey Evid Based Complement Alternat Med 2012 2012 139818 doi:10.1155/2012/139818 22319543 10.1155/2012/139818 PMC3273038

[B3] Arai YC Nishihara M Inoue S Makino I Kampo diagnostic procedure, Fuku shin, could be a useful diagnostic tool for psychopathological patients suffering from chronic pain Evid Based Complement Alternat Med 2013 2013 816216 doi:10.1155/2013/816216 23843883 10.1155/2013/816216 PMC3694461

[B4] Yamakawa J Motoo Y Moriya J Ogawa M Uenishi H Akazawa S Sasagawa T Nishio M Kobayashi J Significance of Kampo, traditional Japanese medicine, in supportive care of cancer patients Evid Based Complement Alternat Med 2013 2013 746486 doi:10.1155/2013/746486 23861712 10.1155/2013/746486 PMC3703882

[B5] Pergolizzi J Ahlbeck K Aldington D Alon E Coluzzi F Dahan A Huygen F Kocot-Kępska M Mangas AC Mavrocordatos P Morlion B Müller-Schwefe G Nicolaou A Pérez Hernández C Sichère P Schäfer M Varrassi G The development of chronic pain: physiological CHANGE necessitates a multidisciplinary approach to treatment Curr Med Res Opin 2013 29 1127 1135 doi:10.1185/03007995.2013.810615 23786498 10.1185/03007995.2013.810615 PMC3793283

[B6] Hijikata Y Miyamae Y Takatsu H Sentoh S Two kampo medicines, jidabokuippo and hachimijiogan alleviate sprains, bruises and arthritis Evid Based Complement Alternat Med 2007 4 463 467 doi:10.1093/ecam/nel105 18227914 10.1093/ecam/nel105 PMC2176138

[B7] Kogure T Tatsumi T Shigeta T Fujinaga H Sato T Niizawa A Effect of kampo medicine on pain and range of motion of osteoarthritis of the hip accompanied by acetabular dysplasia: case report and literature review Integr Med Insights 2011 6 13 17 doi:10.4137/IMI.S7884 22174570 10.4137/IMI.S7884 PMC3236006

[B8] Otsuka K KAMPO-A Clinical Guide to Theory and Practice 2010 Edinburgh: Churchill Livingstone, Elsevier

[B9] Shibata Y Wu J KAMPO Treatment for Climacteric Disorders 1997 Brookline: Massachusetts, Paradigm Publications

[B10] Suzuki Y Goto K Ishige A Komatsu Y Kamei J Antinociceptive effect of Gosha-jinki-gan, a Kampo medicine, in streptozotocin-induced diabetic mice Jpn J Pharmacol 1999 79 169 175 10202852 10.1254/jjp.79.169

[B11] Omiya Y Suzuki Y Yuzurihara M Murata M Aburada M Kase Y Takeda S Antinociceptive effect of shakuyakukanzoto, a Kampo medicine, in diabetic mice J Pharmacol Sci 2005 99 373 380 16314688 10.1254/jphs.fp0050536

[B12] Yamaguchi T Tsujimatsu A Kumamoto H Izumi T Ohmura Y Yoshida T Yoshioka M Anxiolytic effects of yokukansan, a traditional Japanese medicine, via serotonin 5-HT1A receptors on anxiety-related behaviors in rats experienced aversive stress J Ethnopharmacol 2012 143 533 539 doi:10.1016/j.jep.2012.07.007 22819689 10.1016/j.jep.2012.07.007

[B13] Hidaka T Yonezawa R Saito S Kami-shoyo-san, Kampo (Japanese traditional medicine), is effective for climacteric syndrome, especially in hormone-replacement-therapy-resistant patients who strongly complain of psychological symptoms J Obstet Gynaecol Res 2013 39 223 228 doi:10.1111/j.1447-0756.2012.01936.x 22765925 10.1111/j.1447-0756.2012.01936.x

[B14] Ogawa K Kojima T Matsumoto C Kamegai S Oyama T Shibagaki Y Muramoto H Kawasaki T Fujinaga H Takahashi K Hikiami H Goto H Kiga C Koizumi K Sakurai H Shimada Y Yamamoto M Terasawa K Takeda S Saiki I Identification of a predictive biomarker for the beneficial effect of a Kampo (Japanese traditional) medicine keishibukuryogan in rheumatoid arthritis patients Clin Biochem 2007 40 1113 1121 17673196 10.1016/j.clinbiochem.2007.06.005

[B15] Odaguchi H Wakasugi A Ito H Shoda H Gono Y Sakai F Hanawa T The efficacy of goshuyuto, a typical Kampo (Japanese herbal medicine) formula, in preventing episodes of headache Curr Med Res Opin 2006 22 1587 1597 16870083 10.1185/030079906X112769

[B16] Ito N Yabe T Nagai T Oikawa T Yamada H Hanawa T A possible mechanism underlying an antidepressive-like effect of Kososan, a Kampo medicine, via the hypothalamic orexinergic system in the stress-induced depression-like model mice Biol Pharm Bull 2009 32 1716 1722 19801833 10.1248/bpb.32.1716

[B17] Toriizuka K Kamiki H Ohmura NY Fujii M Hori Y Fukumura M Hirai Y Isoda S Nemoto Y Ida Y Anxiolytic effect of Gardeniae Fructus-extract containing active ingredient from Kamishoyosan (KSS), a Japanese traditional Kampo medicine Life Sci 2005 77 3010 3020 15985266 10.1016/j.lfs.2004.12.054

